# Differential effects of desvenlafaxine on hot flashes in women with breast cancer taking tamoxifen: a randomized controlled trial

**DOI:** 10.1038/s41523-024-00668-w

**Published:** 2024-07-17

**Authors:** Yongjoo Kim, Chan-Woo Yeom, Hyun Jeong Lee, Jeong-Hyun Kim, Kwang-Min Lee, Tae-Yong Kim, Han-Byoel Lee, Hoon Kim, Seock-Ah Im, Kyung-Hun Lee, Miso Kim, Wonsik Han, Hyeong-Gon Moon, David Spiegel, Bong-Jin Hahm, Kyung-Lak Son

**Affiliations:** 1 College of Korean Medicine, Sangji University, Wonju, Republic of Korea; 2 Department of Psychiatry, Uijeongbu Eulji Medical Center, Uijeongbu, Republic of Korea; 3 Mental Health Clinic, National Cancer Center, Goyang, Republic of Korea; 4 Department of Psychiatry, Seoul National University Bundang Hospital, Seongnam, Republic of Korea; 5 Mind Lab Place Psychiatry Clinic, Seoul, Republic of Korea; 6 Department of Internal Medicine, Seoul National University Hospital, Seoul, Republic of Korea; 7 Department of Surgery, Seoul National University Hospital, Seoul, Republic of Korea; 8 Department of Obstetrics and Gynecology, Seoul National University College of Medicine, Seoul, Republic of Korea; 9 Department of Obstetrics and Gynecology, Seoul National University Hospital, Seoul, Republic of Korea; 10 Department of Psychiatry and Behavioral Sciences, Stanford University, Stanford, CA USA; 11 Department of Neuropsychiatry, Seoul National University Hospital, Seoul, Republic of Korea; 12 Department of Psychiatry, Dongguk University Ilsan Hospital, Goyang, Republic of Korea

**Keywords:** Randomized controlled trials, Predictive markers

## Abstract

Hot flashes (HF) are a common adverse event of prolonged tamoxifen use in women with estrogen receptor-positive breast cancer, impacting psychiatric health and quality of life. While desvenlafaxine does not interact with tamoxifen, its efficacy and safety in breast cancer patients remain unstudied. This phase 3, four-week, multi-center, three-arm, parallel-group, randomized, double-blind, placebo-controlled trial investigated the efficacy and safety of desvenlafaxine for treating HF in women with breast cancer taking tamoxifen, assessing potential differential effects in patients with psychiatric and inflammatory conditions. Between December 2017 and February 2019, 57 women aged 19 or older, regularly taking tamoxifen as adjuvant therapy, experiencing moderate-to-severe HFs for more than a month, were randomized to receive desvenlafaxine 50 mg/day (D-50), desvenlafaxine 100 mg/day (D-100), or placebo for four weeks. The primary endpoint was the change rate in HF scores over four weeks, with adverse events as a secondary endpoint. Both desvenlafaxine arms demonstrated greater HF score reductions compared to placebo: D-50 (2.20 points/week, 95% CI: 0.71, 3.68) and D-100 (2.34 points/week, 95% CI: 0.92, 3.76). Notably, D-50 arm showed significantly greater efficacy in patients with depression or elevated inflammation. Desvenlafaxine offers an effective and safe treatment regimen for HF in women with breast cancer taking tamoxifen. The presence of depression and inflammation may guide optimal desvenlafaxine dosing. (Trial Registration: ClinicalTrials.gov Identifier: NCT02819921)

## Introduction

Breast cancer is the most prevalent cancer among women, accounting for 24.5% of all female cancer cases^1^. In 2020, 2.26 million women globally were newly diagnosed with breast cancer, and the number is rapidly increasing^1^. Among the various breast cancer subtypes, approximately 80% are characterized by the presence of estrogen receptors (ER-positive)^2^. Tamoxifen, a widely used adjuvant therapeutic agent, has been proven effective in reducing the risk of recurrence of ER-positive breast cancer post-surgery^3^. As tamoxifen functions by inhibiting the binding of estrogen to its receptor, its use can induce menopausal symptoms, such as hot flashes (HFs) in premenopausal women, and exacerbate HFs in postmenopausal women^4,5^.

HFs substantially contribute to psychiatric distress among patients with breast cancer, leading to insomnia, depression, and fatigue, ultimately diminishing their quality of life^6^. Given the significantly improved 5-year survival rate for patients with breast cancer, which now reaches 90%, and the long-term use of tamoxifen (nearly ten years), the effective management of HFs has become an increasingly critical concern.

Conventional hormone replacement therapy for HFs can increase the risk of ER-positive breast cancer recurrence. As a result, there has been growing attention toward non-hormonal therapies, such as antidepressants, to address HFs among patients with breast cancer^7^. While some antidepressants, including paroxetine and sertraline, have been used as alternatives to hormone replacement therapy for HF, they inhibit the metabolism of tamoxifen by the cytochrome P450 2D6 (CYP2D6) enzyme into its active metabolites, potentially reducing tamoxifen’s anticancer effects^8^.

Recently, there has been growing attention to venlafaxine and desvenlafaxine as more appropriate alternatives. Since neither venlafaxine nor desvenlafaxine is metabolized by CYP2D6, no known drug-drug interaction with tamoxifen has been observed^8^. Regarding the treatment of hot flashes in the general female population, while the efficacy of venlafaxine and desvenlafaxine is known to be similar^9^, the incidence of adverse events such as nausea and the corresponding dropout rate have been reported to be lower among those receiving desvenlafaxine compared to venlafaxine^10^. This suggests that desvenlafaxine may be a safer therapeutic option for breast cancer patients taking tamoxifen.

While RCTs have investigated the efficacy and safety of venlafaxine for treating hot flashes in women with breast cancer taking tamoxifen^5^, no previous RCT has explored the efficacy and safety of desvenlafaxine in this population. Current research linking desvenlafaxine to HF management has predominantly focused on non-cancer populations, particularly women undergoing natural menopause. Earlier clinical studies have shown that desvenlafaxine substantially alleviates HFs in postmenopausal women compared to placebo^11–13^. However, no studies have investigated its effects among breast cancer populations.

In this study, we conducted a randomized controlled trial to investigate the efficacy and safety of desvenlafaxine in addressing HFs in women with breast cancer taking tamoxifen. Moreover, we examined potentially differential effects of desvenlafaxine based on psychiatric conditions (e.g., depression and insomnia) and inflammatory processes, as indicated by elevated levels of pro-inflammatory cytokines (e.g., interleukin-1β, interleukin-6, interleukin-8, and tumor necrosis factor-α). It is known that pro-inflammatory cytokines elevate the body temperature set-point in the hypothalamus, precipitating hot flashes^14^. Additionally, IL-1β, IL-6, and TNF-α produce nitric oxide, which dilates blood vessels and contributes to hot flashes^15^. Desvenlafaxine has anti-inflammatory effects, reducing inflammation indirectly by improving depressive symptoms^16^ and directly acting on immune cells to lower cytokine secretion^17^. Therefore, desvenlafaxine’s anti-inflammatory effect may help reduce hot flashes, with baseline inflammation possibly moderating this effect.

## Results

### Patients and Treatment

**Fig. 1:**
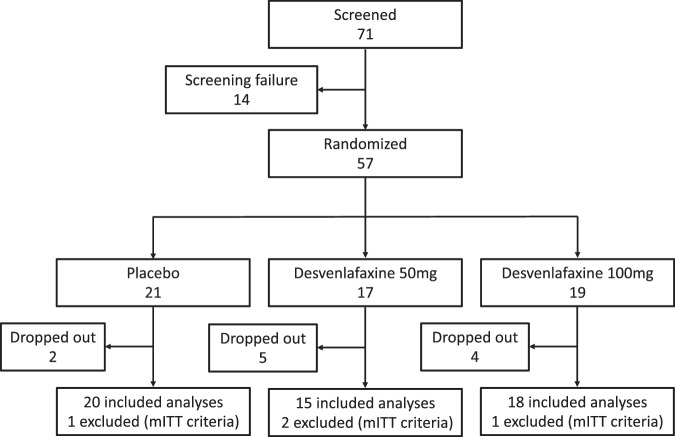
Study enrolment flowchart of women with breast cancer taking tamoxifen. mITT, modified Intention-to-Treat.

Between December 1, 2017, and February 7, 2019, a total of 61 patients were recruited. Of these, four patients did not meet the inclusion criteria. Consequently, 57 patients were enrolled in the study and randomly assigned to the study arms: 17 patients for D-50, 19 for D-100, and 21 for placebo. Of those, four patients did not meet the mITT criteria for study adherence. Therefore, our efficacy analysis using the mITT sample comprised 53 participants, whereas the safety analysis included all 57 participants (Fig. 1).

### Baseline Characteristics

Baseline sociodemographic, behavioral, and medical characteristics were generally similar across all study arms (Table 1). The mean age of participants ranged from 45.80 to 49.72 years. The levels of depressive symptoms and concentrations of inflammatory markers were similar across the study arms at baseline. However, the HF scores distributed differently across the study arms at baseline: 14.30 ( ± 7.94) in placebo, 19.85 ( ± 12.38) in D-50, and 30.02 ( ± 14.91) in D-100 arms.

**Table 1:** Baseline distribution of sociodemographic, psychiatric, behavioral, and inflammatory characteristics, and HFs scores among the mITT study sample (*N* = 53)

	All (*N* = 53)	Placebo (*n* = 20)	Desvenlafaxine 50 mg (*n* = 15)	Desvenlafaxine 100 mg (*n* = 18)	*p* value
Age	47.87 ± 7.87	47.75 ± 7.86	45.80 ± 9.24	49.72 ± 6.52	0.367
BMI	22.70 [21.60;24.80]	22.50 [21.15;23.50]	22.50 [21.45;24.45]	24.25 [22.10;26.30]	0.163
Education					1.000
- High	31 (58.49%)	12 (60.00%)	9 (60.00%)	10 (55.56%)	
- Low	22 (41.51%)	8 (40.00%)	6 (40.00%)	8 (44.44%)	
Occupation					0.153
- Employed	32 (61.54%)	15 (75.00%)	9 (64.29%)	8 (44.44%)	
- Unemployed	20 (38.46%)	5 (25.00%)	5 (35.71%)	10 (55.56%)	
Smoking					1.000
- Non-smoker	51 (98.08%)	19 (95.00%)	14 (100.00%)	18 (100.00%)	
-Smoker	1 (1.92%)	1 (5.00%)	0 (0.0%)	0 (0.0%)	
Alcohol					0.729
- Non-drinker	50 (96.15%)	19 (95.00%)	13 (92.86%)	18 100.00%)	
- Drinker	2 (3.85%)	1 (5.00%)	1 (7.14%)	0 (0.0%)	
PHQ-9	5.00 [3.00;9.00]	4.50 [3.00;7.50]	5.00 [3.50;9.00]	5.00 [1.00;11.00]	0.884
Depression					0.591
- Presence	15 (28.30%)	4 (20.00%)	5 (33.33%)	6 (33.33%)	
- None	38 (71.70%)	16 (80.00%)	10 (66.67%)	12 (66.67%)	
PSQI	9.00 [7.00;11.00]	9.40 ± 2.80	8.73 ± 2.66	9.00 ± 2.89	0.776
Insomnia					0.941
- Presence	27 (50.94%)	11 (55.00%)	7 (46.67%)	9 (50.00%)	
- None	26 (49.06%)	9 (45.00%)	8 (53.33%)	9 (50.00%)	
IL-1β (pg/ml)	0.63 [0.53;0.85]	0.63 [0.56;0.70]	0.63 [0.53;0.83]	0.70 [0.59;1.04]	0.642
IL-6 (pg/ml)	1.64 [1.50;2.06]	1.67 [1.47;2.90]	1.75 [1.50;3.35]	1.60 [1.51;1.70]	0.517
IL-8 (pg/ml)	6.93 [5.50;9.11]	7.19 [5.59;9.11]	7.62 [6.01;13.70]	5.82 [4.93;7.89]	0.184
TNF-α (pg/ml)	8.52 ± 2.94	8.35 ± 2.51	8.41 ± 2.81	8.79 ± 3.57	0.897
HF week score	17.71 [10.71;29.14]	14.30 ± 7.94	30.02 ± 14.91	19.85 ± 12.38	0.001*

*mITT* modified Intention-to-Treat, *BMI* body mass index, *PHQ-9* Patient Health Questionnaire-9, *PSQI* Pittsburgh Sleep Quality Index, *IL* Interleukin, *TNF-α* Tumor necrosis factor alpha, *HF* hot flashes

*denotes *p* < 0.05. *P* values were estimated using Analysis of Variance (ANOVA) for continuous variables and Chi-squared tests for binary or categorical variables.

### Overall efficacy

Overall, we found significant evidence of beneficial effects of desvenlafaxine on HFs in women taking tamoxifen (Supplementary Table 2). Compared to placebo, women in D-50 mg arm showed an accelerated improvement rate in HF scores (β − 2.20, 95% CI − 3.68, −0.71, p = 0.004), corresponding to an additional 2.20-point reduction in HF scores weekly over 4 weeks. Similarly, compared to placebo, women in D-100 arm demonstrated a greater improvement rate in HF scores (β − 2.34, 95% CI − 3.76, −0.92, p = 0.001), equivalent to an additional 2.34-point reduction in HF score weekly over 4 weeks.

**Fig. 2:**
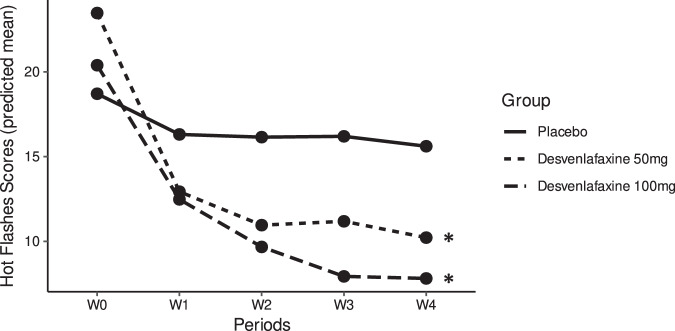
Predicted trajectory plots of mean hot flashes scores across the study arms, placebo, desvenlafaxine 50 mg, and desvenlafaxine 100 mg groups, over the study follow-up period. “*” denotes *p* < 0.05 (reference = placebo). *P* values were estimated using Wald tests derived from linear mixed-effects models.

As shown in Fig. 2 and Supplementary Table 3, three-to-four folds greater percentage reductions were detected in predicted HF scores in the D-50 arm (56.6%) and D-100 arm (61.7%) over 4 weeks, compared to the placebo control (16.6%).

### Interaction and Subgroup Analyses

**Fig. 3:**
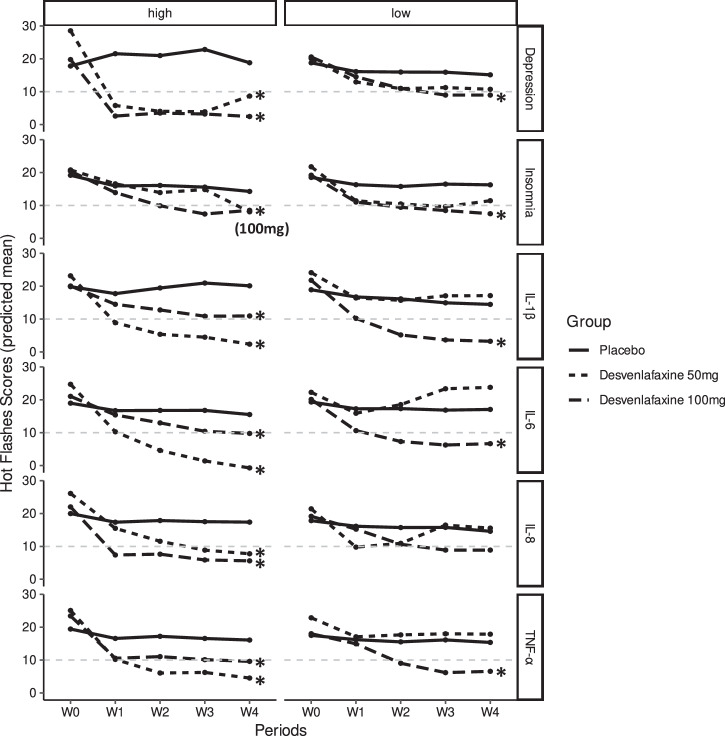
Predicted trajectory plots of mean hot flash scores across study arms: placebo, D-50 mg, and D-100 mg groups, stratified by the proposed psychiatric and inflammatory factors over the follow-up. “*” denotes *p* < 0.05 (reference = placebo). P-values were estimated using Wald tests derived from linear mixed-effects models.

Interaction tests showed evidence of significantly differential effects of desvenlafaxine by levels of depression and pro-inflammatory cytokines (Supplementary Table 4). Overall, the effects of 50 mg desvenlafaxine were generally greater among those with depression or high levels of inflammatory markers than among those without such conditions. For instance, when compared to placebo, the effects estimate of D-50 was 5.64 point-reduction/week (95% CI: 2.09, 9.19) among those with depression, whereas it was 1.22 point-reduction/week (95% CI: -0.43, 2.87) among those without depression (Table 2). Similarly, when compared to placebo, the effects estimate of D-50 was 5.31 point-reduction/week (95% CI: 3.56, 7.06) among those with high levels of IL-6, whereas it was 1.52 point-reduction/week (95% CI: -4.03, 0.99) among those with low IL-6 levels. However, the effects of D-100 were generally consistent across psychiatric and inflammatory factors (Fig. 3).

**Table 2:** Results from subgroup analyses, stratified by proposed psychiatric and inflammatory factors: linear mixed-effects analyses among the mITT study sample (*N* = 53)

Factors	level	Desvenlafaxine 50 mg vs. placebo		Desvenlafaxine 100 mg vs. placebo	
		β (95% CI)	*p* value	β (95% CI)	*p* value
Depression	Presence	−5.64 (−9.19, −2.09)	0.004*	−4.02 (−7.45, −0.59)	0.028*
	None	−1.22 (−2.87, 0.43)	0.151	−2.07 (−3.65, −0.49)	0.011*
Insomnia	Presence	−1.5 (−3.75, 0.75)	0.196	−2.2 (−4.36, −0.04)	0.048*
	None	−1.69 (−3.67, 0.29)	0.098	−2.1 (−3.98, −0.22)	0.031*
IL-1β (pg/ml)	High	−4.95 (−6.7, −3.2)	< 0.001*	−2.53 (−4.14, −0.92)	0.003*
	Low	−0.269 (−2.82, 2.28)	0.837	−3.3 (−5.99, −0.61)	0.018*
IL-6 (pg/ml)	High	−5.31 (−7.06, −3.56)	< 0.001*	−2.08 (−3.83, −0.33)	0.021*
	Low	1.52 (−0.99, 4.03)	0.239	−2.66 (−4.91, −0.41)	0.024*
IL-8 (pg/ml)	High	−3.82 (−5.53, −2.11)	< 0.001*	−2.92 (−4.83, −1.01)	0.003*
	Low	0.182 (−2.72, 3.08)	0.903	−2.01 (−4.36, 0.34)	0.099
TNF-α (pg/ml)	High	−3.84 (−5.4, −2.28)	< 0.001*	−2.13 (−3.68, −0.58)	0.008*
	Low	−0.47 (−3.39, 2.45)	0.753	−2.76 (−5.45, −0.07)	0.047*

*IL* Interleukin, *TNF-α* Tumor necrosis factor alpha, *HF* hot flashes Depression was defined as PHQ-9 score of 9 or above Insomnia was defined as PSQI score of 9 or above.

*denotes *p* < 0.05. *P* values were estimated using Wald tests derived from linear mixed-effects models.

### Dose-optimization exploratory analysis

We further performed exploratory ad hoc analyses using D-100 as the reference group. The results demonstrated that among those with high levels of inflammatory markers, women in the D-50 arm (vs. D-100) showed significantly greater improvements in HF scores, suggesting potentially more beneficial effects of D-50 versus D-100 in this subpopulation (Supplementary Table 5). In contrast, among participants with depression, there was no statistically significant difference in the rate of change in HF scores between the D-50 and D-100 arms, while the directionality of the comparison was consistent with those with elevated inflammation.

### Sensitivity analysis

Our sensitivity analyses using the complete case analysis approach without the LOCF method showed findings consistent with our primary analyses with LOCF. Further sensitivity analyses without baseline adjustment revealed results similar to those of our main analyses with baseline adjustment (Supplementary Tables 6 and 7).

### Safety assessment

Among the 57 participants included in the safety analysis, 23 (35.1%) reported any type of AEs, including 3 cases in the placebo (14.3%), 10 in the D-50 (58.8%), and 10 in the D-100 arms (52.6%). However, none of the 57 participants reported severe AEs during the study period (Supplementary Table 8). The most frequently reported AEs were somnolence, nausea, and vomiting (Supplementary Table 9).

## Discussion

We investigated the efficacy and safety of desvenlafaxine on HFs among women with breast cancer taking tamoxifen and further examined the potentially differential effects of desvenlafaxine by psychiatric and inflammatory factors. We found that both desvenlafaxine 50 mg (D-50) and 100 mg (D-100) showed beneficial effects on HFs over the 4-week study period compared with placebo. We also found significant evidence of differential effects by depression and pro-inflammatory cytokines such as IL-1β, IL-6 and TNF-α, in which the effects of D-50 (vs. placebo) were significantly greater among those with depression or elevated plasma concentrations of such cytokines. In contrast, the effects of D-100 were generally consistent across these factors.

To our knowledge, this is the first study to document the efficacy and safety of desvenlafaxine on HFs among women with breast cancer and the greater benefits of desvenlafaxine 50 mg among those with depression or increased inflammation compared with those without such psychiatric or immunological conditions.

Previous studies have mainly focused on the effects of desvenlafaxine on HFs among women undergoing natural menopause^11–13^. The findings indicated that desvenlafaxine 100 mg vs. placebo was generally effective in addressing HFs among those populations^11–13^. Building upon prior literature, the findings of our present study add to and extend the scientific and clinical knowledge such that the use of desvenlafaxine is effective and safe to address HFs among women with breast cancer taking tamoxifen.

One proposed hypothesis regarding the emergence of HFs during menopause is that the reduction in circulating estrogen levels leads to a corresponding decrease in serotonin concentrations in the blood. This triggers an increase in 5-Hydroxytryptamine 2 A (5-HT2A) receptors within the hypothalamus. This alteration in receptor activity, in turn, leads to a change in the thermoregulatory set point, ultimately resulting in the manifestation of HFs^18^. Based on this proposed process, the primary hypothesis concerning the antidepressant effect on HFs posits that the administration of antidepressants, such as Selective Serotonin Reuptake Inhibitors (SSRIs) and Serotonin and Norepinephrine Reuptake Inhibitors (SNRIs), stabilizes serotonin concentration and receptor numbers. As a result, the central thermoregulatory set point was restored, leading to an improvement in HFs^18^.

We also found that the effects size of D-50 (vs. placebo) was greater in women with underlying depression or increased inflammation than in those without such psychiatric or immunological conditions. In contrast, the effects of D-100 (vs. placebo) were generally consistent across the underlying psychiatric or inflammatory conditions. This may be explained by the interrelationships among depression, inflammation, and HF.

There is a consensus on the reciprocal relationship between depression and HFs^16,19,20^. Karaoulanis et al. highlighted a strong correlation between depression and HFs in naturally menopausal women^19^. Notably, HFs can serve as stressors, potentially contributing to depression. Specific symptoms, such as nocturnal HFs and night sweats, can lead to poor sleep quality, which in turn can exacerbate depression^21^. Pro-inflammatory cytokines are implicated in the development or exacerbation of HFs, particularly in the context of depression^16,20^. Specifically, cytokines such as IL-1β, IL-6, and TNF-α, associated with depression, can induce thermoregulatory abnormalities leading to fever and HFs^14^. Furthermore, these cytokines can cause vasodilation by acting on the blood vessel walls, potentially worsening HFs^19^.

In ad hoc analyses with the D-100 group as a reference, we found a significantly greater effect of D-50, compared to D-100, among those with high-levels of inflammation. However, no significant effects were observed among those with depression when comparing D-50 vs. D-100, whereas the directionality of the comparison was consistent with that in those with elevated inflammation. This may provide clinical evidence to suggest further investigation into the potential role of D-50, compared to D-100, as an optimal dose to address HFs among patients with breast cancer with increased inflammation.

A previous clinical study reported significant effects of D-50 on major depressive disorder^22^. It is possible that the improvement in HFs among such subgroups may be due to the anti-depressive nature of desvenlafaxine and the subsequent reduction in pro-inflammatory cytokines. Consistent findings suggest that pro-inflammatory cytokines decrease following antidepressant treatment for depression^23,24^. Furthermore, antidepressants have been shown to directly influence immune cells such as monocytes, reducing the secretion of pro-inflammatory cytokines such as IL-1β, IL-6 and TNF-α^17,25,26^.

The strengths of our study are as follows. This is the first study to examine the effects of desvenlafaxine on HF among women with breast cancer. While prior research has identified these effects in the general population undergoing natural menopause, no studies have investigated whether such effects exist among women with breast cancer. Furthermore, we incorporated biochemical information and psychiatric measures, and documented differential effects by depression and inflammation status.

The findings of our study should be interpreted considering the following limitations. Initially, our goal was to enroll 303 women with breast cancer taking tamoxifen, with a planned random assignment of 101 women to each study arm. This was based on sample size estimation, which would provide 80% power. However, during the enrollment process, we were able to recruit only 57 participants, limiting the statistical power to detect significant effects. Nonetheless, our study revealed statistically significant effects of desvenlafaxine and modification according to underlying conditions. Further research with larger sample sizes would provide more precise estimates of these associations.

While further research to elucidate the molecular, biological, and psychiatric mechanisms is warranted, the present study contributes insights into the use of desvenlafaxine for HFs in women with breast cancer taking tamoxifen. Furthermore, our findings suggest that, while both desvenlafaxine 50 mg and 100 mg are effective in treating HFs among breast cancer patients, for individuals with underlying increased inflammatory process, desvenlafaxine 50 mg may be an optimal dosage, compared to 100 mg. These clinical insights can inform personalized treatment strategies for women with breast cancer suffering from HFs.

## Methods

### Trial Design

We conducted a phase 3, four-week, multi-center, three-arm, parallel-group, randomized, double-blind, placebo-controlled trial to evaluate the efficacy and safety of desvenlafaxine for moderate-to-severe HFs among women with breast cancer taking tamoxifen regularly (ClinicalTrials.gov Identifier: NCT02819921, registered June 24, 2016). Patients were randomly assigned in a 1:1:1 ratio to receive either desvenlafaxine 100 mg (D-100), desvenlafaxine 50 mg (D-50), or placebo, all administered orally once daily for four weeks. Randomization was performed using a central interactive web-based system and a stratified block randomization method. Stratification factors included age (<50 vs. $\ge$50 years), depression (presence vs. absence), and participating institution.

### Participants

Patients with breast cancer were eligible for enrollment if they were: (a) aged $\ge$19 years, (b) diagnosed with atypical ductal hyperplasia, ductal carcinoma in situ, lobular carcinoma in situ, or invasive adenocarcinoma of the breast (stages I–IV), (c) regularly taking tamoxifen ($\ge$6 times/week) after completing surgery, chemotherapy, and/or radiation therapy, and (d) experiencing moderate-to-severe level of HFs occurring $\ge$14 times/week (on average $\ge$2 times/day) lasting for more than a month. Patients were excluded if they were: (a) pregnant/breastfeeding, (b) had a history of seizure disorder or hepatic/renal dysfunction, (c) taking any hormone therapy (e.g., agents containing estrogen/progesterone/androgens) or taking corticosteroids, or (d) using an antidepressant/gabapentin/pregabalin/clonidine to manage depression and/or HFs.

### Intervention

Participants underwent a 4-week on-therapy period, followed by a 3-day tapering period. For the first week, desvenlafaxine was administered orally at a titration dose of 50 mg daily in both the D-50 and D-100 arms. For the remaining three weeks, desvenlafaxine was administered orally at a dose of 100 mg/day for the D-100 arm (two tablets of 50 mg desvenlafaxine) and 50 mg/day for the D-50 arm (one 50 mg desvenlafaxine plus one placebo pill). Following the on-therapy period, both the D-50 and D-100 arms underwent a 3-day tapering period at a dose of 50 mg/day. The control arm received placebo tablets matching the color and shape of the desvenlafaxine 50 mg pills. Participants in control arm were instructed to take one placebo pill daily during week 1, two pills daily during weeks 2 through 4, and one pill daily during the 3-day tapering period.

### Trial oversight

This trial was funded by Pfizer, and the investigators independently designed and conducted the trial. The trial protocol was approved by the Institutional Review Board of each site (IRB No. H-1606-125-772 from Seoul National University Hospital; NCC2016-0253 from the National Cancer Center; B-1610/365-406 from Seoul National University Bundang Hospital). The trial was conducted in compliance with the principles and guidelines of the Declaration of Helsinki and Good Clinical Practice guidelines of the International Council for Harmonization of Technical Requirements for Pharmaceuticals for Human Use. All participants provided written informed consent before study enrollment, and the confidentiality of their identities was rigorously maintained.

### Primary endpoints

The primary endpoint was the rate of change in HF scores from baseline throughout the follow-up period. The presence and severity of HFs were assessed using the HF diary, a well-validated commonly used scale for evaluating HF symptoms^4,5,27,28^. Participants were instructed to complete the HF diary cards daily, starting from the screening period (one week prior to the study initiation) and continuing until the study termination (at the end of the on-therapy period or loss to follow-up). Each HF event was rated on a scale of “0” for none, “1” for mild, “2” for moderate, “3” for severe, and “4” for very severe. The average weekly HF scores were computed by averaging the daily HF scores (summation of all HF scores within a day) for each week^27^.

Covariate measurements, such as depression, sleep disturbance, and circulating markers of pro-inflammatory cytokines, are provided in Supplementary Table 1.

### Adverse events (AEs) assessment

AEs were monitored via physical examination, vital sign measurements, and electrocardiograms scheduled at baseline and 1st and 4th weeks. The severity of these events was evaluated according to the National Cancer Institute Common Terminology Criteria for Adverse Events version 4.0 (NCI-CTCAE v4.0).

### Ethics approval and consent to participate

The trial received ethics approval from the Institutional Review Board of the participating institutions, with IRB No. H-1606-125-772 from Seoul National University Hospital, IRB No. NCC2016-0253 from the National Cancer Center, and IRB No. B-1610/365-406 from Seoul National University Bundang Hospital. Written informed consent was obtained from all individual participants involved in the trial. The study was conducted in compliance with the ethical principles and guidelines outlines in the Declaration of Helsinki and Good Clinical Practice guidelines established by the International Council for Harmonization of Technical Requirements for Pharmaceuticals for Human Use.

### Statistical analysis

Our initial target sample consisted of 339 patients (113 in each arm). This size was determined to ensure an 80% statistical power, based on previous evidence reporting improvement rates in HF scores for desvenlafaxine 100 mg (mean 80%, SD 72%) versus placebo (mean 47%, SD 79%)^13^. We also considered 10% drop-out rate and employed a two-sided significance level of 0.025, applying Bonferroni correction for multiple testing comparing both D-100 vs. placebo and D-50 vs. placebo^13^.

Efficacy analysis was performed using a Modified Intention-to-Treat (mITT) analysis approach. The mITT sample included all participants who met the following criteria: (a) received the assigned study medication (desvenlafaxine/placebo) for at least five days during the first week, and (b) had their HF score information collected at both the baseline and first-week assessment. Furthermore, to address missing values, we used the Last Observation Carried Forward (LOCF) method.

Based on the above approach, we performed a linear mixed-effects regression analysis, comparing the change rates of HF scores from baseline over the follow-up period between the desvenlafaxine arms (D-100 and D-50) and the placebo. To isolate the effect estimates and 95% confidence intervals (CIs), the interaction terms between time (study week) and treatment assignment were included in the model. To improve the precision of the estimation and account for any potentially unbalanced distribution of HF scores at baseline, we included the baseline HF score as an independent variable in the fixed part of the model. To address the correlation structure induced by repeated measurements of HF scores within individuals during the study period, we included random intercepts and slopes (study week) in the model.

To examine potentially differential effects by depression, insomnia, and inflammatory markers (e.g., IL-1β, IL-6, IL-8, TNF-α), we further performed three-way interaction analyses involving study arm, time, and each proposed factor. If a statistically significant three-way interaction was identified, we conducted subgroup analyses within each proposed factor of effect measure modification.

We performed multiple sensitivity analyses to determine the robustness of the primary findings. First, we employ a complete case analysis approach to manage missing data as an alternative to the LOCF method. Second, we performed a mixed-effects analysis without adjusting for the baseline HF scores.

Safety was evaluated among participants who had taken at least one dose of desvenlafaxine or placebo. Potential differences in the occurrence of mild, moderate, or severe AEs across the arms were tested using Fisher’s exact test with Bonferroni correction to address multiple comparisons.

### Supplementary information

Supplementary Information

Trial Protocol

## Data Availability

The data supporting the findings of this article will be made available upon reasonable request by contacting the corresponding author at klson1@gmail.com.

## Code availability

The codes supporting the findings of this article will be made available upon reasonable request by contacting the corresponding author at klson1@gmail.com.
