# Supplementary material for: Differential effects of desvenlafaxine on hot flashes in women with breast cancer taking tamoxifen: a randomized controlled trial
Source: NPJ Breast Cancer. 2024 Jul 17;10:59. doi: 10.1038/s41523-024-00668-w (PMC11255222; doi:10.1038/s41523-024-00668-w)
Supplement: Supplementary file 1 — Supplementary Information [file 41523_2024_668_MOESM1_ESM.pdf]

Differential Effects of Desvenlafaxine on Hot Flashes in Breast Cancer Patients Taking Tamoxifen: An RCT

Supplementary Information

Supplementary Figure 1. Trajectory plots of crude mean hot flashes scores across the study arms, placebo, desvenlafaxine 50 mg, and desvenlafaxine 100 mg groups, over the study follow-up period

Supplementary Figure 2. Trajectory plots of crude mean hot flash scores across study arms: placebo, D-50 mg, and D-100 mg groups, stratified by the proposed psychiatric and inflammatory factors over the follow-up period

Supplementary Table 1. Covariate Measurements

Supplementary Table 2. Results of linear mixed-effects analysis to investigate the efficacy of desvenlafaxine on HFs among the mITT study sample (N=53)

Supplementary Table 3. Predicted mean hot flash scores across the study arms during the follow-up period

Supplementary Table 4. Interaction tests for effect modification by proposed psychiatric and inflammatory factors: three-way type 3 analyses

Supplementary Table 5. Results from subgroup analyses, stratified by proposed psychiatric and inflammatory factors: Linear mixed-effects analyses among the mITT study sample (N=53) with desvenlafaxine 100 mg as the reference group

Supplementary Table 6. Comparison of the results from the main analysis and sensitivity analyses: Linear mixed-effects analyses to investigate the efficacy of desvenlafaxine on HFs

Supplementary Table 7. Comparison of the results from the main and sensitivity analyses: Predicted mean hot flash scores across study arms during the follow-up periods

Supplementary Table 8. Safety analyses: incidence of adverse events in the ITT study sample (N=57)

Supplementary Table 9. Safety analyses: incidence of adverse events in the ITT study sample (N=57)

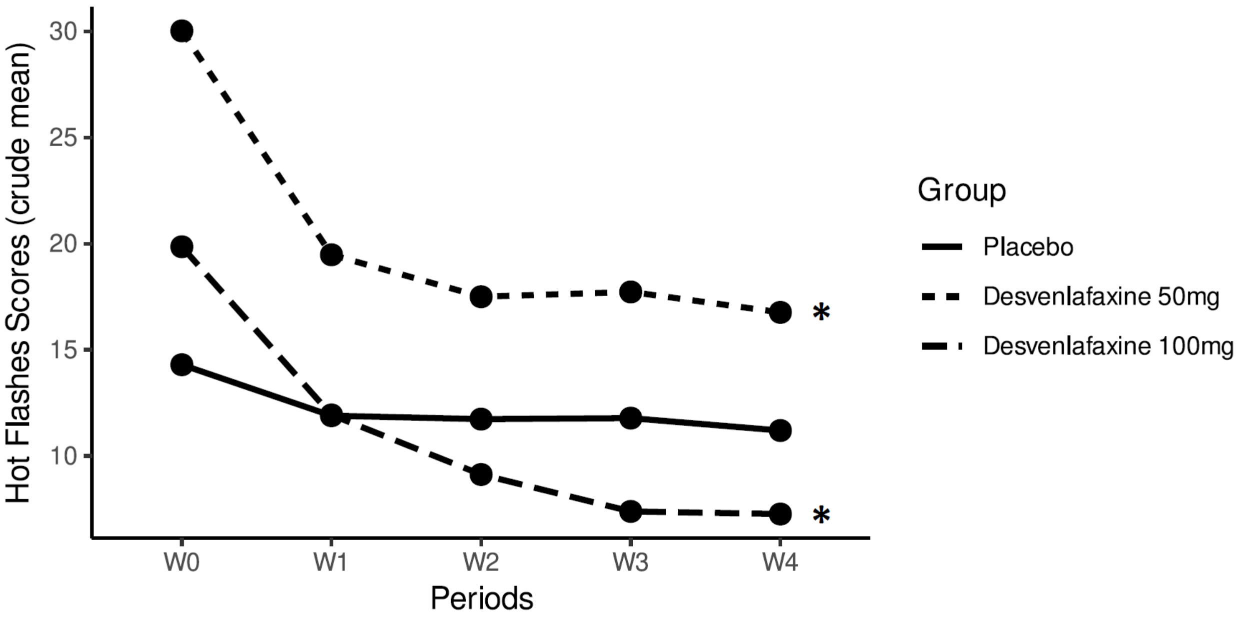

Supplementary Figure 1. Trajectory plots of crude mean hot flashes scores across the study arms, placebo, desvenlafaxine 50 mg, and desvenlafaxine 100 mg groups, over the study follow-up period

" * " denotes p<0.05 (reference = placebo)

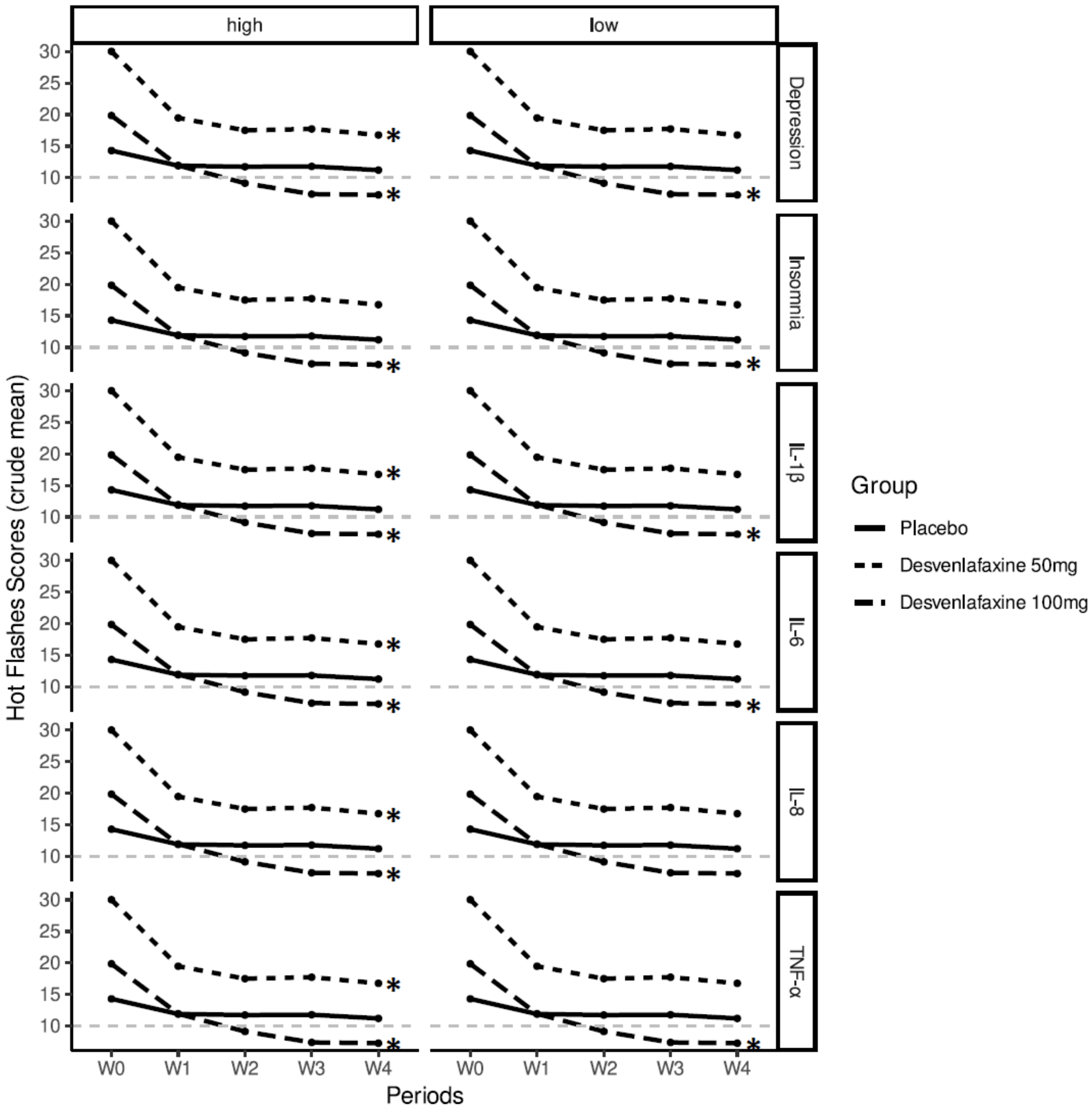

Supplementary Figure 2. Trajectory plots of crude mean hot flash scores across study arms: placebo, D-50 mg, and D-100 mg groups, stratified by the proposed psychiatric and inflammatory factors over the follow-up period

" * " denotes p<0.05 (reference = placebo)

Supplementary Table 1. Covariate Measurements

Covariates	Measurements
1. Psychiatric condition measurements	Depression and sleep quality were considered as the primary psychiatric variables of interest. Information on these variables was collected at baseline and 1st, 2nd, and 4th weeks of the on-therapy period.
1.1. Depression	Depressive symptoms were measured with the Korean-translated Patient Health Questionnaire-9 (PHQ-9), a widely used 9-item screening tool for major depression, which provides a total score ranging from 0 to 27. [1] High-level depressive symptoms were defined as a PHQ-9 score of 9 or above, based on previous research indicating a sensitivity of 0.89–0.91 and a specificity of 0.87–0.95 when this cut-off value was employed to define major depression in comparison with a structured psychiatric interview. [2]
1.2. Sleep disturbance	Sleep quality was assessed using the Korean-translated Pittsburgh Sleep Quality Index (PSQI), a commonly used instrument for evaluating sleep quality. The PSQI score ranges from 0 to 21, whereby higher scores indicate poorer sleep quality and lower scores denote better quality. [7] Insomnia was defined as a PSQI score of 6 or above, considering previous evidence demonstrating a sensitivity of 0.94 and a specificity of 0.84 to identify insomnia or narcolepsy among Koreans using this cut-off value. [8] While a PSQI score of 6 or above is commonly used to signify poor sleep quality in the general population, [7] a threshold of 9 or above is recommended for patients with cancer. [9] Consequently, in our study, we considered a score of 9 or above as indicative of poor sleep quality.
2. Circulating markers of systemic inflammation	Blood samples were drawn from participants at baseline and subsequently sent to a central laboratory for biochemical and immunoassays. Concentrations of circulating pro-inflammatory cytokines, including IL-1β, IL-6, IL-8, and TNF-α, were analyzed using a

	multiplex immunoassay (Merck Millipore, HCYTMAG-60K-PX29, Human Cytokine/Chemokine Magnetic Bead Panel).

* References

1. Kroenke K, Spitzer Robert L. The PHQ-9: A New Depression Diagnostic and Severity Measure. Psychiatric Annals. 2002/09/01/ 2002;32(9):509-515. doi:10.3928/0048-5713-20020901-06

2. Je Yong A, Eun Ran S, Kyung Hi L, Jae Hyun S, Jung Bum K. Standardization of the Korean version of Screening Tool for Depression(Patient Health Questionnaire-9, PHQ-9). Standardization of the Korean Version of Screening Tool for Depression(Patient Health Questionnaire-9, PHQ-9). JOURNAL OF THE KOREAN SOCIETY OF BIOLOGICAL THERAPIES IN PSYCHIATRY. 2013;19(1):47-56.

Supplementary Table 2. Results of linear mixed-effects analysis to investigate the efficacy of desvenlafaxine on HFs among the mITT study sample (N=53)

Terms	β (95% CI)	*p* value
Intercept	4.11 (-2.75 10.97)	0.26
Time (weeks)	-0.63 (-2.22 0.96)	0.47
Placebo	Reference	
Desvenlafaxine 50mg	3.74 (-4.06 11.54)	0.349
Desvenlafaxine 100mg	2.08 (-4.82 8.98)	0.555
Baseline HF scores	0.7 (0.5 0.9)	<0.001 *
Time * Placebo	Reference	
Time * Desvenlafaxine 50mg	-2.2 (-3.69 -0.71)	0.004 *
Time * Desvenlafaxine100mg	-2.34 (-3.75 -0.93)	0.001 *

mITT, modified Intention-to-Treat; HF, hot flashes

Supplementary Table 3. Predicted mean hot flash scores across the study arms during the follow-up period

	Periods	Predicted mean (95% CI)	% change
Placebo	week 0	18.7 (8.9, 28.5)	0
	week 1	16.3 (6.5, 26.1)	-12.8
	week 2	16.2 (6.34, 26)	-13.7
	week 3	16.2 (6.39, 26)	-13.4
	week 4	15.6 (5.80, 25.4)	-16.6
Desvenlafaxine 50mg	week 0	23.5 (13.20, 33.7)	0
	week 1	12.9 (2.68, 23.2)	-44.9
	week 2	11.0 (0.70, 21.2)	-53.3
	week 3	11.2 (0.93, 21.4)	-52.4
	week 4	10.2 (-0.03, 20.5)	-56.5
Desvenlafaxine 100mg	week 0	20.4 (10.5, 30.2)	0
	week 1	12.5 (2.62, 22.3)	-38.8
	week 2	9.67 (-0.18, 19.5)	-52.6
	week 3	7.93 (-1.92, 17.8)	-61.1
	week 4	7.81 (-2.04, 17.7)	-61.7

Supplementary Table 4. Interaction tests for effect modification by proposed psychiatric and inflammatory factors: three-way type 3 analyses

	term	*p* value
Depression	Group	0.280
	Period	0.027
	Depression	0.690
	Baseline HF week score	0.000
	Group * Period	0.000
	Group * Depression	0.093
	Depression * Period	0.091
	**Group * Depression * Period**	**0.050**
Insomnia	Group	0.658
	Period	0.045
	Insomnia	0.494
	Baseline HF week score	0.000
	Group * Period	0.002
	Group * Insomnia	0.965
	Insomnia * Period	0.558
	**Group * Insomnia * Period**	**0.978**
IL-1β	Group	0.684
	Period	0.050
	IL-1β	0.924
	Baseline HF week score	0.000
	Group * Period	0.001
	Group * IL-1β	0.911
	IL-1β * Period	0.891
	**Group * IL-1β * Period**	**0.002**
IL-6	Group	0.744
	Period	0.067
	IL-1β	0.279
	Baseline HF week score	0.000

	Group * Period	0.002
	Group * IL-1β	0.583
	IL-1β * Period	0.000
	**Group:Period:IL-6**	**<0.001**
IL-8	Group	0.790
	Period	0.063
	IL-12	0.674
	Baseline HF week score	0.000
	Group * Period	0.005
	Group * IL-8	0.511
	IL-12 * Period	0.028
	**Group * IL-8 * Period**	**0.053**
TNF-α	Group	0.570
	Period	0.060
	TNF-α	0.896
	Baseline HF week score	0.000
	Group * Period	0.003
	Group * TNF-α	0.990
	TNF-α * Period	0.081
	**Group * TNF-α * Period**	**0.040**

Supplementary Table 5. Results from subgroup analyses, stratified by proposed psychiatric and inflammatory factors: Linear mixed-effects analyses among the mITT study sample (N=53) with desvenlafaxine 100 mg as the reference group

		Desvenlafaxine 50mg vs Desvenlafaxine 100mg		Placebo vs. Desvenlafaxine 100mg	
Factors	level	β (95% CI)	*p* value	β (95% CI)	*p* value
Depression	Presence	-1.63 (-4.84, 1.58)	0.329	4.02 (0.59, 7.45)	0.028 *
	None	0.85 (-0.88, 2.58)	0.338	2.07 (0.49, 3.65)	0.011 *
Insomnia	Presence	0.7 (-1.71, 3.11)	0.572	2.2 (0.04, 4.36)	0.048 *
	None	0.41 (-1.61, 2.43)	0.69	2.1 (0.22, 3.98)	0.031 *
IL-1β (pg/ml)	High	-2.41 (-3.87, -0.95)	0.002 *	2.53 (0.92, 4.14)	0.003 *
	Low	3.03 (0.03, 6.03)	0.05	3.3 (0.61, 5.99)	0.018 *
IL-6 (pg/ml)	High	-3.22 (-5.02, -1.42)	0.001 *	2.08 (0.33, 3.83)	0.021 *
	Low	4.18 (1.71, 6.65)	0.001 *	2.66 (0.41, 4.91)	0.024 *
IL-8 (pg/ml)	High	-0.9 (-2.82, 1.02)	0.36	2.92 (1.01, 4.83)	0.003 *
	Low	2.19 (-0.57, 4.95)	0.124	2.01 (-0.34, 4.36)	0.099
TNF-α (pg/ml)	High	-1.71 (-3.34, -0.08)	0.043 *	2.13 (0.58, 3.68)	0.008 *
	Low	2.29 (-0.61, 5.19)	0.125	2.76 (0.07, 5.45)	0.047 *

IL, Interleukin; TNF-α, Tumor necrosis factor alpha; HF, hot flashes
Depression was defined as PHQ-9 score of 9 or above
Insomnia was defined as PSQI score of 9 or above

Supplementary Table 6. Comparison of the results from the main analysis and sensitivity analyses: Linear mixed-effects analyses to investigate the efficacy of desvenlafaxine on HFs

	Main Analysis: using LOCF and baseline HF adjustment		Sensitivity Analysis 1: using complete case analysis and baseline HF adjustment		Sensitivity Analysis 2: using LOCF, but without baseline HF adjustment	
Terms	β (95% CI)	*p* value	β (95% CI)	*p* value	β (95% CI)	*p* value
Intercept	4.11 (-2.75 10.97)	0.26	4.09 (-2.5 10.68)	0.244	14.07 (6.88 21.26)	0.001 *
Time (weeks)	-0.63 (-2.22 0.96)	0.47	-0.65 (-2.18 0.88)	0.439	-0.63 (-2.22 0.96)	0.469
Placebo	Reference		Reference		Reference	
Desvenlafaxine 50mg	3.74 (-4.06 11.54)	0.349	3.87 (-3.79 11.53)	0.325	14.7 (5.72 23.68)	0.002 *
Desvenlafaxine 100mg	2.08 (-4.82 8.98)	0.555	2.41 (-4.39 9.21)	0.489	5.95 (-2.6 14.5)	0.176
Baseline HF scores	0.7 (0.5 0.9)	<0.001 *	0.7 (0.5 0.9)	<0.001 *		
Time * Placebo	Reference		Reference		Reference	
Time * Desvenlafaxine 50mg	-2.2 (-3.69 -0.71)	0.004 *	-2.32 (-3.91 -0.73)	0.005 *	-2.2 (-3.69 -0.71)	0.004 *
Time * Desvenlafaxine100mg	-2.34 (-3.75 -0.93)	0.001 *	-2.44 (-3.95 -0.93)	0.002 *	-2.34 (-3.75 -0.93)	0.001 *

LOCF: last observation carried forward

HF, hot flashes

mITT, modified Intention-to-Treat; HF, hot flashes

Supplementary Table 7. Comparison of the results from the main and sensitivity analyses: Predicted mean hot flash scores across study arms during the follow-up periods

	Periods	Main Analysis: using LOCF and baseline HF adjustment		Sensitivity Analysis 1: using complete case analysis and baseline HF adjustment		Sensitivity Analysis 2: using LOCF, but without baseline HF adjustment	
		mean (95% CI)	% change	mean (95% CI)	% change	mean (95% CI)	% change
Placebo	week 0	18.7 (8.9, 28.5)	0	18.4 (3.54, 33.29)	0	14.3 (3.1 25.5)	0
	week 1	16.3 (6.5, 26.1)	-12.8	16 (1.14, 30.89)	-13	11.9 (0.702 23.1)	-16.8
	week 2	16.2 (6.34, 26)	-13.7	15.9 (0.98, 30.73)	-13.9	11.7 (0.541 22.9)	-17.9
	week 3	16.2 (6.39, 26)	-13.4	15.9 (1.03, 30.77)	-13.7	11.8 (0.588 23)	-17.6
	week 4	15.6 (5.80, 25.4)	-16.6	15.2 (0.27, 30.05)	-17.7	11.2 (0.0037 22.4)	-21.7
Desvenlafaxine 50mg	week 0	23.5 (13.20, 33.7)	0	23.1 (7.93, 38.28)	0	30 (18.3 41.7)	0
	week 1	12.9 (2.68, 23.2)	-44.9	12.6 (-2.61, 27.73)	-45.6	19.5 (7.79 31.2)	-35.1
	week 2	11.0 (0.70, 21.2)	-53.3	10.3 (-4.95, 25.5)	-55.5	17.5 (5.81 29.2)	-41.7
	week 3	11.2 (0.93, 21.4)	-52.4	10.7 (-4.56, 25.99)	-53.6	17.7 (6.04 29.4)	-41
	week 4	10.2 (-0.03, 20.5)	-56.5	9.5 (-5.83, 24.8)	-58.9	16.8 (5.08 28.4)	-44.2
Desvenlafaxine 100mg	week 0	20.4 (10.5, 30.2)	0	20.1 (5.17, 34.98)	0	19.9 (8.49 31.2)	0
	week 1	12.5 (2.62, 22.3)	-38.8	12.2 (-2.75, 27.06)	-39.5	11.9 (0.572 23.3)	-39.9
	week 2	9.67 (-0.18, 19.5)	-52.6	10.2 (-4.75, 25.18)	-49.1	9.13 (-2.23 20.5)	-54
	week 3	7.93 (-1.92, 17.8)	-61.1	7.3 (-7.74, 22.27)	-63.8	7.39 (-3.97 18.7)	-62.8
	week 4	7.81 (-2.04, 17.7)	-61.7	7.1 (-7.88, 22.12)	-64.5	7.27 (-4.09 18.6)	-63.4

LOCF: last observation carried forward

HF, hot flashes

mITT, modified Intention-to-Treat; HF, hot flashes

Supplementary Table 8. Safety analyses: incidence of adverse events in the ITT study sample (N=57)

	Desvenlafaxine 100mg (N=19)	Desvenlafaxine 50mg (N=17)	Placebo (N=21)	Global *p* value
Any AE	10 (52.6%)	10 (58.8%)	3 (14.3%)	0.009 *
Mild AEs	6 (31.6%)	8 (47.1%)	3 (14.3%)	0.088
Moderate AEs	5 (26.3%)	4 (23.5%)	0 (0.0%)	0.043 *
Severe AEs	0 (0.0%)	0 (0.0%)	0 (0.0%)	1.000

Supplementary Table 9. Safety analyses: incidence of adverse events in the ITT study sample (N=57)

	Desvenlafaxine 100mg (N=19)	Desvenlafaxine 50mg (N=17)	Placebo (N=21)	*p* value
Cardiovascular disorders	2 (10.5%)	0 (0.0%)	1 (4.8%)	0.632
- Hypertension	0 (0.0%)	0 (0.0%)	1 (4.8%)	1.000
- Arrhythmia	1 (5.3%)	0 (0.0%)	0 (0.0%)	0.632
- Palpitation	1 (5.3%)	0 (0.0%)	0 (0.0%)	0.632
Gastrointestinal disorders	2 (10.5%)	4 (23.5%)	0 (0.0%)	0.053
- Dyspepsia	1 (5.3%)	0 (0.0%)	0 (0.0%)	0.632
- Nausea or Vomiting	1 (5.3%)	3 (17.6%)	0 (0.0%)	0.064
- Constipation	0 (0.0%)	2 (11.8%)	0 (0.0%)	0.085
General disorders	5 (26.3%)	2 (11.8%)	0 (0.0%)	0.029 *
- Fatigue	3 (15.8%)	0 (0.0%)	0 (0.0%)	0.056
- Somnolence	2 (10.5%)	2 (11.8%)	0 (0.0%)	0.309
- Edema	1 (5.3%)	0 (0.0%)	0 (0.0%)	0.632
Metabolism and nutrition disorders	2 (10.5%)	2 (11.8%)	2 (9.5%)	1.000
- Hyperlipidemia	2 (10.5%)	2 (11.8%)	2 (9.5%)	1.000
Nervous system disorders	3 (15.8%)	1 (5.9%)	0 (0.0%)	0.142
- Dizziness	1 (5.3%)	0 (0.0%)	0 (0.0%)	0.632
- Headache	2 (10.5%)	1 (5.9%)	0 (0.0%)	0.387
- Tinnitus	1 (5.3%)	0 (0.0%)	0 (0.0%)	0.632
Psychiatric disorders	2 (10.5%)	3 (17.6%)	0 (0.0%)	0.146
- Depression	1 (5.3%)	0 (0.0%)	0 (0.0%)	0.632
- Insomnia	1 (5.3%)	3 (17.6%)	0 (0.0%)	0.064
Renal and urinary disorders	2 (10.5%)	1 (5.9%)	1 (4.8%)	0.828
- Proteinuria	2 (10.5%)	1 (5.9%)	0 (0.0%)	0.387
- High leukocyte levels in the urine	0 (0.0%)	0 (0.0%)	1 (4.8%)	1.000
- Microscopic hematuria	0 (0.0%)	0 (0.0%)	1 (4.8%)	1.000

Reproductive system disorders	1 (5.3%)	0 (0.0%)	0 (0.0%)	0.632
- Vaginal itching	1 (5.3%)	0 (0.0%)	0 (0.0%)	0.632
