# Supplementary material for: Differential effects of desvenlafaxine on hot flashes in women with breast cancer taking tamoxifen: a randomized controlled trial
Source: NPJ Breast Cancer. 2024 Jul 17;10:59. doi: 10.1038/s41523-024-00668-w (PMC11255222; doi:10.1038/s41523-024-00668-w)
Supplement: Supplementary file 2 — Trial Protocol [file 41523_2024_668_MOESM2_ESM.docx]

**Title: Desvenlafaxine for the treatment of hot flashes in women with breast cancer taking Tamoxifen: a randomized, double-blind, placebo-controlled study**

**Protocol Version No. 2.71**

**Principal Investigator:**

Bongjin Hahm, MD, PhD
Department of Psychiatry, Seoul National University Hospital
101, Daehak-ro, Jongno-gu, Seoul 03080, Republic of Korea
Email: hahmbj@gmail.com

**Trial Overview**

| Title | Desvenlafaxine for the treatment of hot flashes in women with breast cancer taking Tamoxifen: a randomized, double-blind, placebo-controlled study |
| --- | --- |
| Principal Investigator | Bongjin Hahm, MD, PhD  Department of Psychiatry, Seoul National University Hospital |
| Funding agency | Pfizer Ltd. (Investigator-initiated trial) |

| Research Aim | To determine whether desvenlafaxine is effective and safe in treating hot flashes in patients with breast cancer taking Tamoxifen compared to placebo. |
| --- | --- |
| Study design | 4-week dosing, multicenter, randomized, double-blind, placebo-controlled trial |
| Study Period | From IRB Approval Date through December 31st, 2022 |
| Study Participants | Patients with breast cancer taking Tamoxifen and have been experiencing moderate or severe hot flashes at least 14 times per week (an average of 2 or more times per day) for a duration of 1 month or longer. |
| Investigational Drugs | Desvenlafaxine (oral administration, once daily, in two doses: 50mg and 100mg) |
| Sample Size | 339 patients with breast cancer (244 from the primary hospital) |
| Vulnerable research participants | Not applicable |
| Trial Design | # 4-week dosing, multicenter, randomized, double-blind, placebo-controlled study  # Arm 1: Desvenlafaxine 100mg group  First week: one tablet of desvenlafaxine 50mg every morning.  Next three weeks: Two tablets of desvenlafaxine 50mg every morning.  Subsequent three days: One tablet of desvenlafaxine 50mg every morning for gradual tapering  # Arm 2: Desvenlafaxine 50mg group  First week: one tablet of desvenlafaxine 50mg every morning.  Next three weeks: One tablet of desvenlafaxine 50mg plus one placebo tablet every morning.  Subsequent three days: One placebo tablet every morning for gradual tapering.  # Arm 3: Placebo group  First week: one placebo tablet every morning.  Next three weeks: Two placebo tablets every morning.  Subsequent three days: One placebo tablet every morning  # During the study period, participants are required to maintain a daily diary recording their hot flashes symptoms. They will also visit the hospital a total of four times, undergoing four questionnaire assessments and two sets of blood and urine tests.  # Survey Questionnaires  - CGI (Clinical Global Impression), adverse event assessment.  - Hot flashes diary (completed daily)  - EORTC-QLQ-CIPN-20 (peripheral neuropathy).  - MINI (screening for psychiatric disorders)  - PHQ-9 (depression), GAD-7 (anxiety), MDQ (mania)  - PSQI (sleep quality)  - MEQ (morningness-eveningness preference)  - MCTQ (circadian rhythm misalignment)  - FACIT-Fatigue (fatigue)  - FACT-B (quality of life)  - BMQ (Medication Belief Scale), BIPQ (Illness Perception Questionnaire).  - MSPSS (Multidimensional Scale of Perceived Social Support)  - BIS (Body Image Scale)  - CDRS (Connor-Davidson Resilience Scale)  # Blood and urine tests  a) Female sex hormones  - Estrogen, estradiol, progestron, lutenizing hormone (LH), follicle-stimulating hormone (FSH), AMH (Anti-Müllerian hormone)  b) Monoamine metabolites & serotonin receptor  - 5-hydroxyindole acetic acid (5-HIAA), 3-methoxy-4-hydroxyphenylglycol (MHPG)  - Platelet 5HT2A receptor  c) Pro & anti-inflammatory cytokines  - Interleukin-1β (IL-1β), IL-6, IL-12, and IL-18, tumor necrosis factor alpha (TNF-α),  - interferon gamma (IFNγ), granulocyte-macrophage colony stimulating factor (GM-CSF)  - IL-4, IL-10, IL-11, IL-13  d) KNDy peptides  - Kisspeptin, neurokinin B, dynorphin  e) Calcitonin gene‐related peptide (CGRP)  f) Serum lipid profile, Urinalysis  g) genetic polymorphism in estrogen receptors (ESR1 PvuII; rs#2234693 and XbaI; rs#9340799 and ESR2-02; rs#4986938) and serotonin transporter gene (SLC6A4; rs#11080121)  h) Urinalysis |
| Efficacy evaluation | # A self-reported hot flush diary completed daily  - The daily occurrence of hot flashes and their severity, rated on a scale from 1 (mild) to 4 (very severe), are recorded together.  - The daily hot flush score is derived by multiplying the frequency of symptoms by their severity. The average score over a week is then computed, and after 4 weeks of treatment, the efficacy of desvenlafaxine in reducing hot flash symptoms is evaluated in terms of both presence and degree of reduction. |
| Safety evaluation | Adverse events, lipid tests, and urine tests |
| Expected impacts and anticipated results | While hot flashes in the general population can often be treated with hormonal therapy, this approach is not viable for breast cancer patients specifically, as the use of hormonal therapy can increase the risk of further cancer progression.  Desvenlafaxine does not affect cancer progression and shows no drug interactions with Tamoxifen, making it safe for use. Consequently, it holds promise as an effective treatment for hot flashes in breast cancer patients on Tamoxifen.  This study is a randomized, double-blind, placebo-controlled trial designed to evaluate the efficacy of Desvenlafaxine as an alternative to hormonal therapy in these patients.  Additionally, through blood tests for estradiol, FSH, and AMH, as well as association analyses with the estrogen receptor gene and serotonin transporter gene, we aim to establish a biological foundation for predicting risk factors and treatment responses to hot flashes in breast cancer patients on Tamoxifen. |

**Research Protocol**

1. **Research title**

Desvenlafaxine for the treatment of hot flashes in women with breast cancer taking Tamoxifen: a randomized, double-blind, placebo-controlled study

1. **Research institutes**

- Seoul National University Hospital, Seoul, Republic of Korea
- Seoul National University Bundang Hospital, Seongnam-si, Gyeonggi-do, Republic of Korea
- National Cancer Center, Goyang-si, Gyeonggi-do, Republic of Korea

1. **Principal investigator and co-investigators**
2. **Principal investigator**

- Bongjin Hahm, MD, PhD, Department of Psychiatry, Seoul National University Hospital, Seoul, Republic of Korea

1. **Co-Investigators**

- Jeong-Hyun Kim, MD, PhD, Seoul National University Bundang Hospital, Seongnam, Republic of Korea
- Hyunjung Lee, MD, PhD, National Cancer Center, Goyang, Republic of Korea
- Kyung-Lak Son, MD, PhD, Department of Psychiatry, Dongguk University Ilsan Hospital, Goyang, Korea
- Seock-Ah Im, MD, PhD, Department of Internal Medicine, Seoul National University Hospital, Seoul, Republic of Korea
- Tae-Yong Kim, MD, PhD, Department of Internal Medicine, Seoul National University Hospital, Seoul, Republic of Korea
- Kyung-Hun Lee, MD, PhD, Department of Internal Medicine, Seoul National University Hospital, Seoul, Republic of Korea
- Miso Kim, MD, PhD, Department of Internal Medicine, Seoul National University Hospital, Seoul, Republic of Korea
- Wonsik Han, MD, PhD, Department of surgery, Seoul National University Hospital, Seoul, Republic of Korea
- Hyeong-Gon Moon, MD, PhD, Department of surgery, Seoul National University Hospital, Seoul, Republic of Korea
- Han-Byoel Lee, MD, PhD, Department of Surgery, Seoul National University Hospital, Seoul, Republic of Korea

1. **Research Coordinator**

- Suyeon Roh, RN, Department of Psychiatry, Seoul National University Hospital, Seoul, Republic of Korea

1. **Investigational Drug Control Pharmacist**

- Hongwon Jang, Seoul National University Hospital Biomedical Research Institute

1. **Funding agency**

- Pfizer Korea, Seoul, Republic of Korea

1. **Study period**

- IRB Approval Date - December 31, 2022

1. **Research target disease**

- Hot flashes in breast cancer patients taking tamoxifen

1. **Background and objectives of the study**

**1) Background**

Tamoxifen is commonly used as adjuvant treatment after surgery and chemotherapy for early and advanced estrogen receptor positive (ER-positive) breast cancers. Tamoxifen blocks the role of estrogen in the body, a common side effect is hot flashes, a symptom of menopause in premenopausal women, and worsening of hot flashes in postmenopausal women. Hot flashes reduce the quality of sleep in patients with breast cancer and make them more tired, thereby reducing their overall quality of life [1].

Hormone therapy using estrogen and progesterone is the most effective treatment for hot flashes. However, in women with breast cancer, these hormonal treatments can be potential risk factors for breast cancer progression and are not commonly used. Therefore, there is a need for alternative hormone therapies to control hot flashes in women with breast cancer taking tamoxifen.

Antidepressants (Selective Serotonin Reuptake Inhibitors, SSRIs; Serotonin and norepinephrine reuptake inhibitors, SNRIs), Gabapentin, and Clonidine have been studied as non-hormonal treatments for hot flashes in women with breast cancer. Among these, venlafaxine (SNRI), Paroxetine (SSRI), and Gabapentin have been reported as promising non-hormonal treatments for hot flashes in patients with breast cancer [2]. However, most of these are accompanied by various side effects such as nausea, fatigue, dry mouth, constipation, drowsiness, and insomnia. Furthermore, paroxetine reduces the anticancer effect of Tamoxifen on drug interactions by affecting Cytochrome P450 2D6 (CYP2D6) metabolism in the liver. Gabapentin had no additive effect on depressive symptoms accompanied by hot flashes in patients with breast cancer. Therefore, there is an urgent need to develop non-hormonal therapies that are effective, have few side effects, and are safe for cancer treatment to improve hot flashes in these patients.

Desvenlafaxine is an SNRI antidepressant that is considered a non-hormonal treatment.

**2) Objectives Of the study**

Desvenlafaxine is the main active metabolite of Venlafaxine, and has fewer side effects and drug interactions than Venlafaxine. In previous studies, desvenlafaxine 100 mg had no effect on CYP2D6 metabolism and no pharmacokinetic drug interactions with tamoxifen [3].

Desvenlafaxine has been shown to reduce the frequency and severity of hot flashes in several randomized controlled clinical trials in postmenopausal women [4]. In these studies, desvenlafaxine, starting at 50 mg and titrating up to 100 mg, was effective in improving menopausal hot flashes by 55-69%, was safe, and had few side effects. Therefore, desvenlafaxine can be considered an effective and safe treatment for hot flashes in patients with breast cancer taking tamoxifen. Presently, no clinical trials have been conducted as in this study in desvenlafaxine.

This was a 4-week, multi-centre, randomized, double-blind, placebo-controlled study of desvenlafaxine 50 mg and 100 mg to determine whether it is effective in reducing the number and severity of hot flashes in breast cancer patients taking tamoxifen.

1. **Investigational drugs**

**1) Product name**

- *Pristiq* (Active ingredient: Desvenlafaxine 50 mg per tablet; Pale pink, square, one side pyramid-shaped, with sustained-release film coating.)

**2) Placebo**

- Starchy tablet, identical in appearance to *Pristiq*

1. **Inclusion criteria, exclusion criteria, number of study participants targeted and rationale for calculation**
2. **Inclusion criteria**

- Women, age 19 years or older
- Diagnosed with atypical ductal hyperplasia, ductal carcinoma in situ, lobular carcinoma in situ, or invasive breast adenocarcinoma stages I through IV.
- Taking tamoxifen regularly (6+ times/week) following the termination of surgery, chemotherapy, and/or radiation therapy due to breast cancer
- Moderate to severe hot flashes occurring 14+ times/week (on average, 2+ times/day) lasting for more than a month.

1. **Exclusion Criteria**

- Pregnant or breastfeeding
- History of seizure or hepatic/renal dysfunction
- Receiving any hormone therapy (including estrogen-/progesterone-/androgens-containing agents) or taking corticosteroids
- Use of antidepressants such as gabapentin, pregabalin, or clonidine to address depression and/or hot flash symptoms.
- Currently suffering from or having a history of severe psychiatric symptoms, such as hallucinations and delusions, mania, and high suicide risk

1. **Number of participants targeted and rationale for calculation**

This study aimed to determine whether the use of desvenlafaxine in patients suffering from hot flashes reduces its symptoms.

The primary endpoint was defined as the percentage reduction in hot flush symptoms = (symptom score at week 4–symptom score at a week before treatment) / symptom score at a week before treatment), and the following assumptions were made to calculate the number of participants to fulfil the primary outcome of the study.

① Level of significance, α = 0.025

The study was divided into three groups (50 mg, 100 mg, placebo), and a type 1 error was defined as 0.025 to determine whether there was a significant difference between the Desvena 50 mg treatment group vs. the placebo group and the 100 mg treatment group vs. placebo group.

② Type II error (β) is set to 0.20, and the power of the test is maintained at 80%.

③ The hypotheses in this study are as follows (two-tailed test):

- Null hypothesis: There is no difference in the rate of reduction of hot flashes between the 50 mg vs. placebo groups or between the 100 mg vs. placebo groups.
- Alternative hypothesis: There is a difference in the rate of reduction of hot flashes between the 50 mg versus placebo or 100 mg versus placebo groups.

The number of participants was calculated based on a comparison of the desvenlafaxine 100 mg group with the placebo group. In a similar study conducted previously on menopausal women complaining of hot flashes, the mean reduction rate was 0.8 and standard deviation (SD) 0.72 when desvenlafaxine 100 mg was administered, and in the case of the placebo group, the average reduction rate was 0.47 and standard deviation 0.79 [5]. The number of participants in the study that achieved 80% power at a 2.5% significance level was 101 in each arm, for a total of 303. The number of participants was planned to be 339 to account for a dropout rate of 10 %.

1. **Study participant recruitment plan**

Participants will be recruited by posting the [Notice of Recruitment of Research Subjects] in the outpatient departments of the haematology oncology and integrated care center, surgery, obstetrics/gynecology, and mental health clinic. In addition, participants will be recruited by posting a recruitment announcement on “https://cafe.naver.com/uvacenter/158559", the representative internet community of the Breast Cancer Patients' Association.

The principal investigator and funding agency will not exclude patients from participation in this study based on race or socioeconomic status. If the selection criteria for this study were met, every effort will be made to allow as many patients as possible to participate in this study.

1. **Study methods**
2. **Specific research methods**

|  | Baseline | Medication Period | | End of Medication | Tapering period |
| --- | --- | --- | --- | --- | --- |
| Period | T0 | T1 | T2 | T3 | 3days |
| Week | -1w | 0w | 1w | 4w | 4w + 3days |
| Day |  | 1 | 8 | 29 | 29–31 |
| Permissible range of visits |  | ±3 days | ±3 days | ±3 days |  |
| Informed consent | O |  |  |  |  |
| Randomization |  | O |  |  |  |
| Study Drug Administration |  | O | O |  |  |
| Medical record check | O |  |  |  |  |
| Physical Examination |  | O | O | O |  |
| Hot flashes diary | O | O | O | X |  |
| Self-reported questionnaires | O | O | O | O |  |
| Blood/urine tests |  | O |  | O |  |
| Assessment of adverse events |  | O | O | O |  |
| Medication adherence evaluation |  |  | O | O |  |
| Concomitant medication check | O | O | O | O |  |

This was a 4-week dosing, multicenter, randomized, double-blind, placebo-controlled trial.

Participants will be randomized to receive desvenlafaxine or placebo for a total of 4 weeks + 3 days, maintain a daily hot flashes diary, and have a total of 4 clinic visits for four questionnaire assessments and two blood and urine tests. Height, weight, BMI, and blood and urine tests will be performed at T1 and T3.

The participants will complete an informed consent form at the initial visit, and an initial questionnaire assessment will be administered. If participants did not know the exact date of menstruation in the menstrual history assessment section and the date of breast cancer diagnosis in the cancer-related information section, the following are written: if participants remember only the month, write 15 days (e.g., October ?day 2017 -> October 15, 2017), and if participants remember the beginning, middle, and end of the month instead of the exact date, write 5, 15, and 25 days, respectively (e.g., early October 2017 -> October 05, 2017).

Afterwards, the participants are advised to maintain a hot flashes diary without medication for one week to assess the baseline status before medication. During the same period, patients will be randomized into the desvenlafaxine 100 mg, 50 mg, or placebo group by a third-party institution unrelated to the study (Medical Research Collaborating Center).

From one week after study registration, each of the three groups will be administered a dose of desvenlafaxine or placebo for four weeks. To reduce the adverse effects of abrupt discontinuation in the desvenlafaxine 100 mg group, there will be a 3-day tapering period with one tablet of desvenlafaxine 50 mg or one tablet of placebo.

During the administration period, the patients will complete a hot flashes diary and medication compliance assessments. After participating in the study, patients will receive a hot flashes diary form to record for 1 week, 1 week, and 3 weeks, at visits of -1, 0, and 1 week, and the data written by the patient during that period will be included in the case report form of the previous periods. During the above visits, a physical examination and assessment for adverse events and medication adherence will be performed.

Self-reported questionnaire assessments will be administered at visits 1 week prior to study enrollment and 0, 1, and 4 weeks after study enrolment. Moreover, blood and urine tests will be performed at 0 and 4 weeks after study enrollment.

1. **Control group setting and randomization method**

Participants who provided written informed consent and fulfilled all inclusion and exclusion criteria for enrollment will be randomized in a 1:1:1 ratio to the desvenlafaxine 100 mg arm, desvenlafaxine 50 mg arm, and placebo arm.

A randomization table will be created to assign the participants to one of the three arms using a stratified block randomization method, and web-based allocation will be made according to the randomization table. The stratification factors are age (< 50 and >50 years), presence of depression (defined as a PHQ-9 score of 9 or more), and institution (Seoul National University, Seoul National University Bundang Hospital, National Cancer Center). The randomization table and web-based randomization will be managed and operated by the Medical Research Collaborating Center (MRCC) of Seoul National University/Seoul National University Hospital, which is independent of the study.

If a serious adverse reaction occurs in a participant after participating in the study, they will be withdrawn from the study and unblinded; in this case, the investigator will unblind the participant through a third-party institution (Medical Research Collaborating Center) that manages and operates the randomization.
